# Diffuse large B-cell lymphoma in the new era: prognostic tools for mapping risk

**DOI:** 10.1007/s00277-025-06686-3

**Published:** 2025-10-20

**Authors:** Andrea Duminuco, Salvatore Scarso, Vittorio Del Fabro, Laura Anastasia Caruso, Gaia Stanzione, Francesco Di Raimondo, Giuseppe Alberto Palumbo, Calogero Vetro

**Affiliations:** 1 Hematology with BMT Unit, A.O.U. “G. Rodolico-San Marco”, Via Santa Sofia, Catania, 78–95123 Italy; 2 Department of Infectious, Tropical Diseases and Microbiology, IRCCS Sacro Cuore Don Calabria Hospital, Negrar di Valpolicella, Italy; 3 Faculty of Medicine and Surgery, “Kore” University of Enna, Enna, Italy; 4 Dipartimento di Scienze Mediche Chirurgiche e Tecnologie Avanzate “G.F. Ingrassia”, University of Catania, Catania, Italy; 5 Hematology and BMT Unit, Hospital of Bolzano (SABES-ASDAA), Teaching Hospital of Paracelsus Medical University (PMU), Bolzano, Italy

**Keywords:** Diffuse large b-cell lymphoma, Prognostic models, Overall survival, Progression-free survival, New perspectives

## Abstract

Diffuse large B-cell lymphoma (DLBCL) is clinically and biologically heterogeneous. R-CHOP remains the frontline standard, with polatuzumab-R-CHP conferring a subgroup-dependent progression-free survival gain, yet early relapse and primary refractoriness persist. Classical risk indices, especially IPI and revised versions, retain benchmark value but only partially capture adverse biology. This review integrates established and emerging tools: multivariable clinical scores (e.g., NPI, GELTAMO), geriatric models (GPI), host-status metrics (HALP, GNRI), PET-derived tumor burden and dissemination (TMTV, TLG; IMPI), immune-microenvironment signatures, and health-system markers (diagnosis-to-treatment interval). We summarize genetic predictors (e.g., CD79B/PIM1) and dynamic molecular response via ctDNA (early/major molecular response), and appraise interim PET as prognostic but not treatment-directive outside trials. In relapsed/refractory disease, second-line age-adjusted IPI and pre-transplant PET inform autologous transplantation candidacy, while randomized trials establish CD19 CAR-T as superior to salvage chemoimmunotherapy in early relapse or primary refractory settings; bispecific antibodies and antibody–drug conjugates expand options post-CAR-T or for transplant-ineligible patients. On the other side, CNS risk assessment is best approached with CNS-IPI refined by site and genotype; prophylaxis remains individualized given mixed efficacy signals. Overall, risk-adapted, biologically driven care should report NCCN-IPI (alongside IPI) in all patients and incorporate imaging burden, genomics, and ctDNA where feasible.

## The landscape of diffuse large B-cell lymphoma (DLBCL)

Diffuse large B-cell lymphoma (DLBCL) is the most common histologic subtype of non-Hodgkin lymphoma (NHL), representing approximately 30% of all cases in adults. It is a biologically and clinically heterogeneous group of aggressive B-cell malignancies, unified under the umbrella of DLBCL not otherwise specified (NOS) in current classifications [1]. DLBCL can arise in nodal or extranodal sites and typically presents with rapidly enlarging masses, systemic symptoms, and elevated serum lactate dehydrogenase (LDH). Gene expression profiling (GEP) has defined two major subtypes based on cell of origin (COO): germinal center B-cell-like (GCB) and activated B-cell-like (ABC), which are associated with different outcomes and responses to therapy [2]. While GEP remains the gold standard for COO classification, it is not routinely performed in clinical settings. Instead, immunohistochemical algorithms based on CD10, BCL6, and IRF4/MUM1 are widely used as practical surrogates [3].

The 2022 WHO classification and the International Consensus Classification (ICC) have further refined the spectrum of large B-cell lymphomas, recognizing molecularly and clinically distinct variants, including LBCL with IRF4 rearrangement, LBCL with 11q aberration, and EBV-positive DLBCL. These entities, along with rare forms such as primary effusion-based lymphoma, intravascular LBCL, and fibrin-associated DLBCL, present unique diagnostic challenges and often require tailored approaches. Standard diagnostic work-up includes excisional lymph node biopsy, extended immunophenotyping, and fluorescence in situ hybridization (FISH) testing for MYC rearrangement, with additional testing for BCL2 and BCL6 rearrangements when MYC is positive. PET/CT imaging, comprehensive bloodwork, and viral serologies complete the initial staging [4–6]. In cases of deep-seated lymphadenopathies, nodes not easily accessible surgically, or patients not eligible for excisional biopsy, an ultrasonography-guided core-needle biopsy represents a safe and effective alternative. Recent multicenter studies in Italy have demonstrated that this approach provides adequate tissue for histopathological, immunophenotypic, and molecular analyses, with minimal complications, making it a practical option in routine clinical practice [7–9].

Despite advances in treatment, most notably the addition of rituximab to CHOP chemotherapy, a substantial subset of patients fails to achieve durable remission. Early relapses and primary refractory disease remain associated with poor outcomes. In recent years, the therapeutic landscape of DLBCL has rapidly evolved with the advent of novel agents such as chimeric antigen receptor T-cell (CAR-T) therapies, bispecific antibodies, and antibody-drug conjugates (ADCs) [10]. These innovative treatments have significantly improved prognosis in the relapsed/refractory setting and are increasingly being investigated in earlier lines of therapy.

However, the optimal integration of these agents into frontline treatment remains a major clinical challenge. Traditional clinical risk models, such as the International Prognostic Index (IPI) discussed below, provide limited ability to identify patients at high risk of treatment failure. As a result, there is growing interest in the development and application of refined prognostic tools that incorporate clinical, biological, and imaging data to inform therapeutic decisions more effectively, in the field of lympho- and myeloproliferative neoplasms, aiming to ensure the best stratification [11–16].

This review aims to provide an updated and comprehensive overview of the prognostic landscape in DLBCL, from well-established models to emerging molecular and functional predictors. Emphasis will be placed on identifying high-risk diseases in the context of modern therapeutic options, to inform risk-adapted, biologically driven treatment strategies.

## Therapeutic approaches to DLBCL

### Newly diagnosed diseases

#### Limited-stage disease (Stage I–II)

Patients with limited-stage DLBCL (Ann Arbor stage I–II) generally have an excellent prognosis, with reported 10-year overall survival rates of at least 70–80% [17]. Historically, abbreviated chemotherapy (3–4 cycles of R-CHOP) followed by involved-site radiotherapy (RT) has been widely used, as consolidation RT has consistently been demonstrated to improve progression-free and overall survival [18]. More recently, PET-adapted strategies, such as those evaluated in the LYSA/GOELAMS study, have demonstrated that omitting RT in non-bulky patients who achieve a complete metabolic response after 4 cycles yields similarly excellent outcomes [19, 20]. Additionally, the FLYER trial demonstrated in young patients with favorable-risk disease that a shortened regimen of four cycles of R-CHOP plus two additional rituximab doses is non-inferior to six full cycles, with fewer adverse events [21]. Overall, these data support the feasibility and safety of treatment de-escalation in selected limited-stage DLBCL, with RT reserved for bulky or metabolically active residual disease. Furthermore, modern reviews emphasize that PET-guided reduction of both chemotherapy and radiotherapy is an evolving and promising strategy [18].

#### Advanced-stage disease (Stage III–IV)

For advanced-stage DLBCL, six cycles of R-CHOP every 21 days (R-CHOP-21) remains the standard first-line regimen across most international guidelines. Multiple large randomized trials have confirmed its efficacy, with progression-free survival (PFS) and overall survival (OS) generally in the ~ 70–80% and ~ 80–90% ranges, respectively (e.g., R-CHOP arms showing 2-year PFS ≈ 70–75% and 2-year OS ≈ 85–89%) [22].

Attempts to improve outcomes using dose-dense strategies such as R-CHOP-14 have not yielded survival benefits; in a 1,080-patient phase III trial, PFS and OS were similar to R-CHOP-21, with higher rates of febrile neutropenia and infections on R-CHOP-14 [23]. Similarly, dose-adjusted EPOCH-R (DA-EPOCH-R), despite theoretical advantages in biologically aggressive subtypes, was not superior to R-CHOP in the CALGB/Alliance 50,303 study and produced more grade ≥ 3 toxicities [24]. A significant advancement in frontline therapy has come from the integration of targeted agents. The phase III POLARIX trial evaluated polatuzumab vedotin in place of vincristine within R-CHOP (Pola-R-CHP) in 879 untreated patients with IPI 2–5: at ~ 28 months’ median follow-up, 2-year PFS improved to 76.7% with Pola-R-CHP vs. 70.2% with R-CHOP, with no difference in 2-year OS; subgroup analyses (forest plot) suggested greater benefit in patients >60 years, ABC subtype, and IPI ≥ 3 [25]. Toxicity was broadly comparable: peripheral neuropathy occurred at similar frequencies in both arms (~ 53% any grade), while febrile neutropenia was higher with Pola-R-CHP (~ 14% vs. ~ 8%) [26]. Finally, patient outcomes with Pola-R-CHP may vary according to molecular subtypes (e.g., ABC vs. GCB, double-/triple-hit lymphomas, MYD88/CD79B mutations), highlighting the value of integrating molecular and genetic markers into therapeutic decision-making.

Despite a modest absolute benefit, Pola-R-CHP is now an accepted frontline option for selected higher-risk patients in contemporary practice guidelines [27].

In elderly or frail patients, standard regimens like R-CHOP may be associated with increased toxicity. Here, alternative approaches with reduced dose (R-miniCHOP) or the use of liposomal doxorubicin, can mitigate the risk [28]. A phase II study demonstrated that a regimen combining liposomal doxorubicin, vinblastine, and dacarbazine, followed by consolidation radiotherapy, resulted in a complete response rate of 90% in older adults with advanced-stage DLBCL or classical Hodgkin lymphoma, associated with manageable toxicity [29]. New prognostic tools can be helpful also in this case to balance benefits and risks.

### Refractory/recidivant DLBCL

The treatment of relapsed or refractory (R/R) DLBCL has undergone a paradigm shift with the advent of immunotherapeutic strategies. Yet, high-dose chemotherapy followed by autologous stem-cell rescue (HDT/ASCR) remains a potentially curative option for a select group with chemosensitive disease [30]. Retrospective series report ~ 50% 5-year PFS and ~ 60–65% 5-year OS after autologous stem cells transplant (ASCT), and in a population-based cohort of 1,039 R/R DLBCL patients, the 4-year OS from first relapse was 28% overall and 51% among those who actually underwent autoHCT. Outcomes were far worse when the relapse occurred within 12 months of diagnosis or when the disease was primary refractory (4-year OS ~ 13%) [31]. Pre-transplant FDG-PET status is strongly prognostic: patients who are PET-negative by Deauville 1–3 before ASCT have substantially superior post-ASCT PFS/OS compared with PET-positive cases [32].

CD19-directed CAR T-cell therapy has redefined the standard for high-risk R/R DLBCL. In ZUMA-1, axicabtagene ciloleucel achieved an ORR of 83% with CR 58%, and at 5 years, the estimated OS was 42.6% and disease-specific survival 51%. Two randomized phase III trials established CAR-T superiority in second line for early relapse/primary refractory disease: ZUMA-7 (axi-cel) improved response rates (ORR 83% vs. 50%; CR 65% vs. 32%) and event-free survival vs. salvage chemoimmunotherapy plus ASCT, while TRANSFORM (lisocabtagene maraleucel) showed higher CR (74% vs. 43%) and markedly longer PFS (NR vs. 6.2 months) [33, 34]. In contrast, BELINDA (tisagenlecleucel) did not improve EFS over standard care [35].

For transplant-ineligible patients in second line, the single-arm PILOT study of lisocabtagene maraleucel reported high activity and supported use in this setting [36]. In following lines, TRANSCEND NHL-001 reported an overall response rate of 73% and CR 53% with liso-cel and durable remissions on follow-up [37].

Bispecific antibodies provide effective options post-CAR-T or for frail patients. Subcutaneous epcoritamab produced ORR ~ 63% with CR ~ 39–40% in EPCORE NHL-1, with durable responses on longer follow-up, including meaningful activity in primary-refractory and prior-CAR-T subgroups [38]. Fixed-duration IV glofitamab achieved ORR 52% and CR 39% in a pivotal phase II trial, with many CRs maintained beyond treatment [39].

Cytokine release syndrome (CRS) and immune effector cell-associated neurotoxicity syndrome (ICANS), associated with long term immunosuppression and impaired immune system are potential complications that must be promptly recognized and treated [40–44].

Among targeted antibodies, tafasitamab plus lenalidomide (L-MIND) yielded ORR 57.5% (CR 41%) with median PFS 11.6 months and median OS 33.5 months at 5-year analysis, with deeper, more durable responses when used earlier [45]. The antibody–drug conjugate polatuzumab vedotin with bendamustine-rituximab (Pola-BR) improved CR (40% vs. 18%), PFS (10 vs. 4 months), and OS (12 vs. 5 months) versus BR alone in transplant-ineligible R/R DLBCL, while loncastuximab tesirine demonstrated ORR ~ 48% (CR ~ 24%) with median OS ~ 9–10 months in the single-arm LOTIS-2 study [46, 47].

Together, these data support a risk-adapted strategy: prioritize cellular therapies for early relapsers or primary refractory disease, while leveraging bispecific antibodies and other targeted agents for patients relapsing after CAR-T or those ineligible for intensive approaches.

A brief therapeutic summary is reported in Table 1.

**Table 1:** Available treatment for frontline and relapsing/refractory diffuse large B-cell lymphoma (DLBCL)

Setting	Regimen (trials)	Population	Results	Comments
Front-line treatments	R-CHOP-21 (standard)	Advanced-stage, unselected	2-year PFS ~ 70–75%; 3-year ~ 65% OS ~ 80–90% at 2–3 yrs	Well tolerated, standard of care
	Polatuzumab-R-CHP (POLARIX)	Untreated DLBCL, IPI ≥ 2	2-yr PFS 76.7% vs. 70.2%; 5-yr 64.9% vs. 59.1% (HR 0.77) OS: No significant difference with R-CHOP; trend HR 0.85 (NS)	Similar safety to R-CHOP; fewer subsequent therapies
Relapsed/refractory	HDT/ASCR (pre-CAR-T era)	Chemo-sensitive relapse or second CR group	5-yr DFS ~ 48% 5-yr OS ~ 63%	Benefit in late relapse; limited eligibility
	Axi-cel (ZUMA-1; ZUMA-7)	≥ 2 prior lines or early relapse/IPI ≥ 3	ORR 83%, CR 58% EFS median 8 mo vs. 2 mo (ZUMA-7) 5-yr OS ~ 43%	Superior to HDT/ASCR in early relapse; high CRS/ICANS
	Liso-cel (TRANSCEND; TRANSFORM)	≥ 2 lines (TRANSCEND) or early relapse/ref (TRANSFORM)	ORR 73%, CR 53% 1-yr PFS ~ 44%; median PFS NR in CR responders; TRANSFORM CR 74% vs. 43%, median PFS NR vs. 6 mo 1-yr OS ~ 58%; TRANSFORM 18-mo OS ~ 73% vs. 54%	Superior efficacy in early relapse; tolerable toxicity
	Tisa-cel (JULIET; BELINDA)	≥ 2 lines (JULIET); second-line early relapse (BELINDA)	ORR 53%, CR 39% Median PFS 3 mo; OS 11 mo	BELINDA: no benefit over standard therapy
Post–CAR-T or CAR-T/transplant-ineligible	Epcoritamab (EPCORE NHL-1)	R/R DLBCL after ≥ 2 lines, including prior CAR-T	ORR 63%, CR 39% Median PFS ~ 4 mo OS NR; 6-mo PFS ~ 44%	Effective post-CAR T relapse; manageable CRS/hematological
	Glofitamab	R/R population, fixed-duration (12 cycles)	ORR 52%, CR 39% Median PFS ~ 5 mo; 12-mo OS ~ 50% CR responders: 67% in CR at 18 mo	Durable remissions in CR; prior CAR-T patients responded
	Tafasitamab and Lenalidomide (L-MIND)	Transplant-ineligible R/R DLBCL	ORR 58%, CR 40% Median PFS 12 mo; OS 34 mo 36-mo OS: 81% in CR; 34% in PR	Hematologic toxicity; better responses when used earlier
	Polatuzumab + BR	R/R DLBCL, transplant-ineligible	CR 40% vs. 18% Median PFS 10 mo vs. 4 mo OS 12 mo vs. 5 mo	Improves outcomes across COO and double-expressor status
	Loncastuximab Tesirine (LOTIS-2)	Heavily pretreated R/R DLBCL	ORR 48%, CR 24% Median PFS ~ 5 mo; OS ~ 10 mo	Active in refractory settings; manageable toxicity

*PFS *progression-free survival, *OS *overall survival, *HR *hazard ratio, *NS *not significant, *CR *complete response, *IPI* international prognostic index, *DFS *disease-free survival, *EFS *event-free survival, *R/R* recidivant/refractory, *ORR *overall response rate, *mo *months, *NR *not reached, *PR *partial response

## Standard prognostic models and biological features in DLBCL

### International prognostic index (IPI)

The International Prognostic Index (IPI) is a clinical tool used to stratify risk in patients with DLBCL. It incorporates five factors: age >60 years, elevated LDH, Ann Arbor stage III/IV, Eastern Cooperative Oncology Group (ECOG) performance status >1, and >1 extranodal site of disease. Patients are assigned to four risk groups (low, low-intermediate, high-intermediate, high) based on the number of adverse factors, with corresponding 5-year overall survival rates ranging from approximately 88% (low) to 54% (high) [1, 48].

The IPI remains the standard for clinical risk stratification and is widely used in clinical trials and practice. However, it does not reliably identify patients with very high-risk disease, especially in the rituximab era, nor does it account for underlying biological heterogeneity such as cell-of-origin (COO) or high-risk molecular subtypes [1, 48].

Cell-of-origin classification (GCB vs. ABC) and high-risk molecular subtypes (e.g., double-hit lymphomas with MYC and BCL2/BCL6 rearrangements) are independent prognostic factors. These biological markers are not included in the IPI but are associated with inferior outcomes and may guide therapeutic decisions. Combining IPI with molecular features (e.g., MYC/BCL2 expression, COO) improves risk stratification and identifies patients who may benefit from alternative or intensified therapies [1, 49].

### Revised IPI (R-IPI)

The Revised International Prognostic Index (R-IPI) is a simplified adaptation of the original IPI for diffuse large B-cell lymphoma (DLBCL) in the rituximab era. It uses the same five clinical factors as the original IPI: age >60 years, elevated LDH, Ann Arbor stage III/IV, ECOG performance status >1, and >1 extranodal site. However, the R-IPI consolidates risk groups into three categories: very-good (0 factors), good (1–2 factors), and poor (3–5 factors). This model provides improved clinical utility by more clearly distinguishing patients with excellent outcomes from those with intermediate or poor prognosis, with 4-year overall survival rates of approximately 94%, 79%, and 55% for the very good, good, and poor groups, respectively [50].

Compared to the original IPI, which stratifies patients into four risk groups, the R-IPI better reflects survival outcomes in patients treated with R-CHOP. Still, neither index identifies a subgroup with long-term survival below 50% in the rituximab era [1].

### National comprehensive cancer network international prognostic index

The NCCN-IPI (National Comprehensive Cancer Network International Prognostic Index) is a revised prognostic tool for DLBCL that improves risk stratification compared to the original IPI. The NCCN-IPI refines age categories, weights LDH levels more granularly, and specifically considers involvement of high-risk extranodal sites (bone marrow, CNS, liver/gastrointestinal tract, lung), in addition to Ann Arbor stage and ECOG performance status. Patients are assigned to four risk groups (low, low-intermediate, high-intermediate, high) based on a point system, with the high-risk group showing a 5-year overall survival of approximately 33–38%, which is lower than the high-risk group in the original IPI, thus better identifying patients with poor prognosis [51, 52]. In pooled analyses of over 2,100 rituximab-treated DLBCL patients, the NCCN IPI demonstrated superior discriminatory ability (C-index ~ 0.632) compared with both the IPI and R-IPI. Five-year OS ranged from approximately 92% in the low-risk group to 49% in the high-risk group.

Current consensus in the medical literature is that while the NCCN-IPI is the most accurate clinical risk model for DLBCL in the rituximab era, optimal risk stratification will require integration of molecular and genetic features alongside clinical parameters [53].

### Cell-of-origin and high-risk molecular subtypes

These models don’t incorporate molecular features such as cell-of-origin (GCB vs. ABC) or high-risk genetic subtypes (e.g., double-hit lymphomas with MYC and BCL2/BCL6 rearrangements). These biological factors are recognized as independent prognostic markers.

COO classification, historically, was central for risk stratification and therapeutic decision-making. Gene expression profiling distinguishes two major molecular subtypes: GCB and ABC [54]. The ABC subtype is associated with inferior outcomes compared to GCB, with 3-year progression-free survival rates of approximately 40–50% versus 75% for GCB, and is characterized by chronic B-cell receptor signaling and NF-κB pathway activation. GCB DLBCL expresses genes typical of germinal center B cells, such as BCL6 and EZH2, and generally has a more favorable prognosis [55].

Immunohistochemistry-based algorithms (e.g., Hans algorithm) are commonly used in clinical practice to approximate COO, but gene expression profiling remains the gold standard for molecular classification [1, 55]. Recent genomic studies have further refined DLBCL taxonomy, identifying genetic subtypes such as MCD (MYD88/CD79B), BN2 (BCL6/NOTCH2), N1 (NOTCH1), and EZB (EZH2/BCL2), each with distinct pathogenic mechanisms and prognostic implications. MCD and N1 subtypes are associated with poor prognosis, while BN2 and EZB subtypes have more favorable outcomes [56].

High-risk molecular subtypes include DLBCLs with MYC and BCL2 and/or BCL6 rearrangements (“double-hit” or “triple-hit” lymphomas), now classified as high-grade B-cell lymphoma (HGBCL-DH/TH) by the World Health Organization. These cases, predominantly of GCB origin, are associated with aggressive clinical behavior and poor outcomes after standard R-CHOP therapy, often warranting consideration of more intensive regimens [1, 57]. Additionally, a “dark zone signature” (DZsig) identifies a poor-prognosis subgroup within GCB-DLBCL, overlapping with but not limited to double-hit cases [57].

## Beyond classical stratification

Several alternative prognostic scores and models for DLBCL risk stratification have been developed and published over the years, beyond the standard IPIs and NCCN-IPI.

### Nomogram prognostic index (NPI)

The NPI integrates clinical variables (age, ECOG performance status, Ann Arbor stage, LDH, extranodal involvement), biologic markers (BCL2 and CD5 expression, B symptoms), and peripheral blood counts (absolute lymphocyte and monocyte counts). In a 1,406-patient cohort, it separated four risk groups with a concordance index of 0.794, exceeding IPI (0.759) and NCCN-IPI (0.750), and maintained discrimination on external validation [58].

### GELTAMO integrative score

Built from age, ECOG performance status, stage, bulky disease, β2-microglobulin, red cell distribution width, and the lymphocyte/monocyte ratio, this model identified an ultra–high-risk subset among 992 R-CHOP–treated patients, with 5-year OS 24% and PFS 19%, compared with 59% and 45% using the R-IPI for an analogous group—indicating sharper separation at the adverse tail of risk [59].

### Geriatric prognostic index (GPI) and older patients

For patients ≥ 70 years, the GPI combines functional status (activities of daily living), comorbidity burden (Charlson Comorbidity Index), age, sex, albumin, stage, ECOG performance status, and LDH. External validation showed 2-year OS of 95% (low risk), 65% (intermediate), and 44% (high), with superior discrimination versus IPI, R-IPI, and NCCN-IPI (C-index 0.710 vs. 0.583–0.670), supporting its use for geriatric tailoring and counseling [60]. In a cohort of 185 patients (median age 75), 5-year OS and PFS were 50.2% and 44.6%. R-CHOP yielded superior OS/PFS versus attenuated regimens despite more grade ≥ 3 neutropenia. Independent adverse predictors included age ≥ 75, high LDH, B-symptoms, stage III/IV, neutrophilia, and low lymphocyte/monocyte ratio [61].

### HALP and GNRI

Simple nutrition/inflammation indices also carry prognostic information. A HALP score (hemoglobin × albumin × lymphocytes/platelets) ≤ 26.17 and a GNRI ≤ 99.17 were independently associated with inferior overall survival in a 201-patient series (e.g., HALP HR 2.32, *p* = 0.004), underscoring the clinical relevance of nutritional status in DLBCL; cut-offs require harmonization before routine adoption [62].

### International metabolic prognostic index (IMPI)

PET/CT-based models incorporating metabolic tumor volume and dissemination metrics refine risk beyond clinical indices in multiple cohorts. IMPI exemplifies this approach: it stratifies outcomes using FDG-PET–derived tumor burden and spread, shows added prognostic value, and can delineate groups with markedly inferior survival, yet lacks broad prospective validation and has not entered standard practice given dependencies on imaging acquisition and segmentation standardization [63, 64].

### Prognostic immune risk score

Leveraging CIBERSORT-derived estimates of tumor-infiltrating immune cells, this immune score robustly partitions risk. In a 956-patient study, high immune risk was associated with inferior 5-year overall survival (HR 3.99 in the training cohort; 2.17 in validation). A nomogram combining this score with clinical variables yielded a C-index of 0.76, exceeding the performance of the IPI and R-IPI [65].

### Diagnosis-to-Treatment interval (DTI) — population registry

In a real-world Ontario cohort (*n* = 9,446), shorter DTI signaled worse biology and inferior survival. Patients starting therapy within 30 days of diagnosis had poorer OS than those treated at 30–60 days (HR 0.72, 95% CI 0.67–0.78) or >60 days (HR 0.78, 95% CI 0.71–0.85); median DTI was 37 days. Effects were independent of distance to the center and socioeconomic variables. Very short DTI likely reflects urgent disease behavior; trials with long screening may under-represent these patients. Overall, DTI operates as a prognostic variable complementing IPI/NCCN-IPI [66].

### Baseline PET tumor burden (TMTV/TLG) — GOYA

From 1,305 PET-evaluable, untreated DLBCL patients, baseline total metabolic tumor volume (TMTV) and total lesion glycolysis (TLG) independently predicted outcomes after R-CHOP-like therapy. ROC cut-offs were 366 cm³ (TMTV) and 3,004 g (TLG). High TMTV was linked to shorter PFS (multivariable HR 1.71, 95% CI 1.35–2.18) and worse OS; SUVmax was non-prognostic. Combining TMTV with IPI or COO sharpened separation (e.g., 5-year PFS 74.3% for low-IPI/low-TMTV vs. 49.0% for high-IPI/high-TMTV) [67].

### Genetic model for early progression

Sequencing 32 genes in 145 DLBCLs identified CD79B and PIM1 mutations, enriched in early progression, plus elevated LDH as independent predictors. A three-variable model (LDH, CD79B, PIM1) achieved AUROC 0.771 and outperformed IPI and MCD/Cluster-5 assignments; findings were externally verified. Pharmacologic assessment suggested sensitivity of mutant cases to BTK (ibrutinib) and pan-PIM (AZD1208) inhibitors, with BCL2 co-inhibition (venetoclax) enhancing apoptosis in double-expressor contexts [68].

### CtDNA molecular response during R-CHOP (EMR/MMR)

Across 217 patients, pretreatment ctDNA was detectable in 98% and prognostic. Early molecular response (EMR, ≥ 2-log drop after cycle 1) and major molecular response (MMR, ≥ 2.5-log drop after cycle 2) independently stratified outcomes beyond IPI and interim PET. In validation, EMR/MMR were associated with higher 24-month EFS (EMR 83% vs. 50%; MMR 82% vs. 46%). These dynamic thresholds function as a quantitative response score usable alongside clinical indices [69].

### Multi-R-signature deep learning (PET-based ensemble)

An ensemble of pre-trained CNNs generates lesion-level “R-signatures” from cropped 2D PET ROIs, then fuses them with clinical/PET covariates (age, Ann Arbor stage, bulky disease, SUVmax, TMTV) to predict PFS/OS; models were trained in one center and validated in another (*n* = 449 PET scans). On the external set, the combined model reached C-index ≈ 0.77 for both PFS and OS, outperforming single-feature baselines and supporting accurate survival risk stratification for newly diagnosed DLBCL. The pipeline selects representative lesion slices via k-means on simple texture/shape features, fine-tunes multiple CNNs, ensembles their output probabilities as multi-R-signatures, and feeds them, together with PET metrics and clinical factors, into conventional survival learners [70].

### Ultrasound-PET/CT nomogram for refractory DLBCL: a single-center experience

A logistic nomogram integrating ultrasound signs and clinical indices predicted refractory disease after standard chemoimmunotherapy in 140 newly diagnosed patients. Blurred lymph-node margins on ultrasound (OR 18.24; *p* < 0.001) and IPI (OR 3.13; *p* = 0.051) were retained, yielding an AUC of 0.835 with 85.5% sensitivity and 79.5% specificity; calibration after internal validation showed close agreement between predicted and observed risk. The model operationalizes readily available baseline data (sono-morphology plus IPI) to flag patients at high risk of primary refractoriness before treatment completion [71].

#### DFR-signature (multimodal PET/CT deep-features + AutoML)

A deep network fuses PET and CT; transfer-learned encoders extract 1,000 deep features per case to build a PET/CT deep-features radiomic signature (DFR). Auto-Machine Learning selects/stack-ensembles features, and the signature is combined with clinical covariates (including NCCN-IPI) in Cox models. Among 369 patients from two centers (7:3 split), the combined model achieved C-indices of 0.784/0.739 for PFS and 0.831/0.782 for OS in training/validation, respectively, and improved risk stratification over clinical factors alone. The study demonstrates complementary anatomic-metabolic information from fused PET/CT and automation to stabilize model building [72].

### Machine-learning GEP survival model

Using GSE10846 (train, *n* = 233) and GSE23501 (test, *n* = 64), the authors identified a 4-gene adverse cluster (TNFRSF9, BIRC3, BCL2L1, G3BP2) and then trained random-forest–based survival models that integrate clinical data, COO, the 4-gene cluster, and expression of 50 genes. The final model delivered C-index 0.84 in training and 0.79 in testing, outperforming stage and COO, and enabling individualized survival prediction from biopsy transcriptomes plus routine variables [73].

### 6-tsRNA serum signature (liquid biopsy)

A six-tsRNA classifier built via random forest/PCA and modeled with Cox regression on an internal RT-qPCR cohort (*n* = 100) identified high-risk patients with markedly inferior OS (HR 6.66; 95% CI 2.83–15.68; *p* = 0.0006), with confirmation in an external cohort (*n* = 160). Integrated with IPI, the signature improved prognostic precision and also supported early response monitoring: an ≳ 1.06-fold decrease after one cycle (“early molecular response”) associated with better outcomes. The panel distinguished DLBCL from healthy controls with an AUC of 0.882, positioning circulating tsRNAs as non-invasive biomarkers for detection, dynamic response assessment, and prognosis [74].

### Interim evaluation

Interim PET, typically performed after 2–4 cycles of immunochemotherapy (most commonly R-CHOP), has been extensively studied for its prognostic value in DLBCL.

Interim PET can predict progression-free and overall survival, with negative interim PET scans associated with favorable outcomes and positive scans indicating higher risk of relapse or progression. Both visual (Deauville score) and quantitative (ΔSUVmax, qPET) methods have been evaluated, with quantitative approaches generally providing superior prognostic discrimination compared to visual assessment alone [75–78]. For example, a reduction in SUVmax of ≥ 66% after two cycles is associated with improved survival, while less reduction identifies poor responders [75, 79].

Meta-analyses confirm that interim PET has a high negative predictive value (often >80%) for progression, but the positive predictive value is more variable and less robust, limiting its utility for guiding risk-adapted therapy outside of clinical trials. In 80 uniformly treated patients (median follow-up 3.36 years), 24 relapsed. Interim PET after 2–4 cycles stratified risk: 2-year PFS was 50.0% for PET-positive vs. 86.4% for PET-negative (*p* = 0.0012; positive predictive value (PPV) 57.1%, negative predictive value (NPV) 75.8%). End-of-treatment PET further sharpened prognosis: 2-year PFS 25.0% for PET-positive vs. 84.7% for PET-negative (*p* < 0.0001; PPV 75.0%, NPV 75.0%) [80].

Current consensus, as described in the medical literature, is that while interim PET is prognostic, treatment modification based solely on interim PET findings is not recommended in routine practice; its use is primarily for risk stratification and clinical trial design [1, 80].

### Second-line setting

Efficacy of salvage therapy correlates with the second-line age-adjusted IPI, and outcomes in R/R DLBCL are shaped by response to initial therapy, relapse timing, and feasibility of high-dose therapy with autologous stem-cell rescue (HDT/ASCR) [31, 81]. In a population-based cohort of 1,039 recidivant/refractory DLBCL patients treated with anthracycline-based chemoimmunotherapy, 244 (23%) had relapsed disease that was re-treated [31]. The 4-year OS was 28% across all salvage approaches, rising to 51% among patients who underwent HDT/ASCR. Prognosis was markedly better with delayed relapse: those relapsing >12 months from diagnosis had a 47% 4-year OS, versus 13% in patients with a transient response or primary refractory disease. Pre-transplant PET further refines risk; positive PET after second-line therapy predicts poor post-transplant outcomes following HDT/ASCR [82].

In R/R DLBCL after R-CHOP (single-center study, *n* = 31), a three-factor score combined time-to-relapse (TTR), LDH ratio (to ULN), and absolute lymphocyte count at relapse (ALC-R). LDH ratio >3 and short TTR independently predicted inferior PFS; low ALC-R added adverse impact. TLL index (Time to relapse–LDH ratio–Lymphocyte count at relapse) stratified 2-year PFS to 100%, 68.6%, and 4.8% across low/intermediate/high-risk groups and predicted limited benefit from ASCT in the highest-risk tier, supporting early alternative strategies [83].

Finally, in 16 CAR-T recipients, higher baseline ctDNA (mean allele frequency ≥ 1%) predicted inferior PFS (median 86.5 days vs. not reached; HR 6.3) and OS (median 219 days vs. not reached; HR 4.1). An early >1.5-log ctDNA decline by day 7–10 correlated with metabolic response at week 4–6 PET; patients lacking this drop rarely responded. ctDNA thus offers a simple, tumor-agnostic biomarker to anticipate early CAR-T outcomes [84].

Collectively, these models sharpen discrimination, particularly at the extremes (very high-risk biology, older/frail patients) or when imaging/omics are available, and can inform supportive care (nutrition), geriatric adjustments, and trial stratification. However, only some have undergone external validation, calibration varies across settings, and none has supplanted IPI or NCCN-IPI as the routine clinical standard. These multi-domain models (clinical, imaging, genomics, host factors) best refine risk, but IPI/NCCN-IPI remain the standard clinical anchor. In a registry of 5,126 newly diagnosed DLBCL patients treated with immunochemotherapy, 13 IPI-like models were compared. All predicted survival, but NCCN-IPI showed the most consistent discrimination; its high-risk 5-year OS (33.4%) matched the original validation. One albumin-based model performed similarly (C-index 0.693) but not better. Models with only three risk groups and without age performed poorly (fit, discrimination, calibration) [48].

A visual representation of new models is reported in Table 2.

**Table 2:** Summary of reported models and scores mentioned in the text

Model/Topic	Domain	Variables/Inputs	Primary Outcomes	Performance and Key Results
Nomogram Prognostic Index (NPI)	Clinical + biologic + hematology	Age; ECOG; Ann Arbor; LDH; extranodal sites; B-symptoms; BCL2; CD5; ALC; AMC	OS (risk-stratified into 4 groups)	C-index 0.794 (IPI 0.759; NCCN-IPI 0.750); external validation maintained
GELTAMO Integrative Score	Clinical + lab	Age; ECOG; stage; bulky; β2-microglobulin; RDW; lymphocyte/monocyte ratio	OS; PFS	High-risk: 5y OS 24%, PFS 19% vs. R-IPI 59%/45%
Geriatric Prognostic Index (GPI)	Geriatric clinical	ADL; Charlson; age; sex; albumin; stage; ECOG; LDH	2y OS: low 95%, int 65%, high 44%	C-index 0.710 (> IPI/R-IPI/NCCN-IPI 0.583–0.670)
HALP & GNRI	Nutrition and inflammation	HALP = Hb×Alb×ALC/PLT; GNRI = 1.489×Alb + 41.7× (W/ideal W)	OS	HALP ≤ 26.17 h 2.32 (*p* = 0.004); GNRI ≤ 99.17 adverse
International Metabolic Prognostic Index	PET/CT (metabolic)	Baseline MTV; dissemination metrics (± age/stage)	OS; PFS	Adds beyond clinical indices; delineates very poor-risk groups
Prognostic Immune Risk Score	Immune microenvironment (CIBERSORT)	Inferred TIIC composition; + clinical nomogram	5y OS	HR 3.99 (train); 2.17 (validation); nomogram C-stat 0.76 (> IPI/R-IPI)
Diagnosis-to-Treatment Interval	Timing/health services	Days diagnosis→treatment start	OS	< 30 d worse OS; HR 0.72 (30–60d), 0.78 (>60 d) vs. < 30d
TLL Index (TTR–LDH ratio–ALC at relapse)	Clinical‑lab	Time to relapse; LDH/ULN; ALC at relapse	PFS; ASCT benefit prediction	2y PFS: 100%/68.6%/4.8% (low/int/high)
Baseline PET tumor burden	PET metrics	TMTV; TLG; ± IPI/COO	PFS; OS	High TMTV HR 1.71 for PFS; cut-offs: 366 cm³ (TMTV), 3,004 (TLG)
Genetic model for early progression	Genetics + LDH	LDH; CD79B; PIM1	Early progression risk	AUROC 0.771; >IPI and cluster assignments
ctDNA molecular response (EMR/MMR) during R‑CHOP	ctDNA dynamics	EMR ≥ 2‑log drop (C1); MMR ≥ 2.5‑log drop (C2)	24 m EFS	EMR 83% vs. 50%; MMR 82% vs. 46%
ctDNA at CAR‑T	ctDNA + cellular therapy	Baseline MAF ≥ 1%; early > 1.5‑log decline d7–10	PFS; OS; metabolic response	High baseline MAF → worse PFS/OS; early decline predicts response
Older adults (≥ 70 y)	Clinical (geriatrics)	Age ≥ 75; LDH; B‑symptoms; stage III/IV; neutrophilia; low LMR	5y OS 50.2%; PFS 44.6%	R‑CHOP > attenuated regimens (↑ neutropenia)
Multi‑R‑signature deep learning	Deep learning on PET + clinical	Lesion‑level CNN sigs; age; stage; bulky; SUVmax; TMTV	PFS; OS	External C‑index ≈ 0.77 (PFS/OS); >single feature baselines
Ultrasound–PET/CT nomogram (refractory risk)	Ultrasound and clinical	Blurred nodal margins; IPI	Refractory disease risk	AUC 0.835; Sens 85.5%; Spec 79.5%
DFR‑signature (PET/CT deep‑features + AutoML)	Radiomics + AutoML	1,000 fused PET/CT deep features; + NCCN‑IPI	PFS; OS	C‑index (train/val): PFS 0.784/0.739; OS 0.831/0.782
Machine‑learning GEP survival model	Transcriptomics + clinical	4‑gene adverse cluster; 50‑gene set; COO; clinical	Survival prediction	C‑index 0.84 (train), 0.79 (test); >stage/COO
6‑tsRNA serum signature (liquid biopsy)	Circulating small RNAs	Six tsRNAs; early molecular response (≥ 1.06× decrease)	OS; detection (AUC)	HR 6.66 (OS); AUC 0.882; EMR linked to outcomes
Interim & end‑of‑treatment PET (prognostic)	PET response (visual & quantitative)	Deauville; ΔSUVmax; qPET	PFS; OS	Interim 2y PFS 50% vs. 86%; End‑PET 25% vs. 84.7%; high NPV, variable PPV
Second‑line predictors & ASCT outcomes	R/R (clinical + imaging)	Second‑line aaIPI; initial response; relapse timing; pre‑ASCT PET	4y OS: 28% overall; 51% with ASCT; 13% if early relapse/primary refractory	Pre‑ASCT PET positivity → poor post‑ASCT outcomes

*LDH *lactate dehydrogenase, *ALC* absolute lymphocyte count, *AMC* absolute monocyte count, *OS* overall survival, *PFS *progression-free survival, *RDW *Red Cell Distribution Width, *ADL* Activities of Daily Living, *Hb *hemoglobin, *Alb *albumin, *PLT *platelets, *W *weight, *MTV *metabolic tumor volume, *TIIC* tumor infiltrating immune cells, *HR *hazard ratio, *TTR *time-to-treatment, *ULN* upper limit normal, *ASCT *autologous stem cells transplant, *TMTV *total metabolic tumor volume, *COO *cell of origin, *TLG *total lesion glycolysis, *EMR *early molecular response, *MMR *major molecular response, *EFS *event-free survival, *MAF *minor allele frequency, *LMR* lymphocyte/monocyte ratio, *AUC* area under the curve, *NPV* negative predictive value, *PPV* positive predictive value, *aaIPI* age-adjusted IPI

#### Risk stratification and management of central nervous system involvement in DLBCL

Central nervous system (CNS) relapse in DLBCL is an uncommon but often fatal complication, with poor prognosis even when treated. To better identify patients at risk, the CNS International Prognostic Index (CNS-IPI), developed by the German DSHNHL group, combines six clinical parameters: age >60 years, elevated LDH, ECOG performance status >1, advanced stage (III–IV), more than one extranodal site, and involvement of the kidneys or adrenal glands. Based on these criteria, patients are stratified into three risk categories with distinct 2-year CNS relapse rates: low (0–1 factors, ~ 0.8%), intermediate (2–3 factors, ~ 3.9%), and high risk (4–6 factors, ~ 12%). Kidney and adrenal involvement emerged as strong site-specific predictors of CNS dissemination. Although the independent effect of the absolute number of extranodal sites varies across studies, “>1 extranodal site” was retained for practicality in the model [85].

Further retrospective analyses confirm that the absolute number of extranodal sites correlates with CNS relapse risk; for example, patients with >2 extranodal locations had a 3-year cumulative CNS relapse incidence of ~ 15% [86]. Certain anatomical presentations display marked CNS tropism, most notably primary testicular DLBCL, and breast involvement has also been associated with excess CNS risk in multi-centre series [87, 88]. In testicular cases, prophylactic measures frequently include contralateral testis/scrotal irradiation (commonly 25–30 Gy; many experts recommend ≥ 30 Gy) and CNS-directed agents such as methotrexate [87, 89].

Molecular features also refine risk prediction: the ABC-like cell of origin subtype and MYD88 and/or CD79B mutations are independently associated with increased CNS relapse risk, even among patients with low or intermediate CNS-IPI scores. Recent studies suggest that integrating these variables with CNS-IPI improves predictive accuracy, identifying subgroups with up to 15% two-year CNS relapse rates [90]. Double expression of MYC and BCL2 proteins, particularly in the ABC subtype, is also linked to higher CNS relapse risk [91].

The role of CNS prophylaxis remains controversial. Observational data consistently show no clear benefit from intrathecal (IT) methotrexate, and large multi-center series and propensity-matched analyses likewise fail to demonstrate a reliable advantage for intercalated or consolidative high-dose intravenous methotrexate (HD-MTX); toxicity and R-CHOP delays are non-trivial. IT methotrexate given with each chemotherapy cycle has long been used, but several retrospective comparisons suggest systemic HD-MTX may confer superior protection, particularly for parenchymal disease [92]. When employed, HD-MTX is commonly administered at 3–3.5 g/m² with leucovorin rescue, frequently on day 15 of R-CHOP-21 and can be integrated safely in selected patients with careful supportive measures (hydration, urine alkalinization, renal/hepatic monitoring) [93]. Flow cytometry of cerebrospinal fluid is recommended to improve diagnostic accuracy when CNS involvement is suspected, and lumbar puncture should be considered in high-risk individuals or those with neurological symptoms [94]. For leptomeningeal disease, combination IT chemotherapy (e.g., methotrexate and/or cytarabine) plus systemic HD-MTX remains standard practice in many centres. Current guidance endorses individualized prophylaxis for patients with high CNS-IPI scores or disease involving high-risk extranodal sites (e.g., testis, kidney, adrenal glands, breast), recognizing that breakthrough CNS relapses still occur despite prophylaxis) [95, 96].

## Conclusion

In DLBCL, the NCCN-IPI remains the cornerstone for clinical risk stratification. However, maximal prognostic discrimination is achieved by integrating clinical parameters with molecular features, imaging-derived metrics, and dynamic biomarkers such as ctDNA. Interim PET should be used for risk stratification rather than guiding automatic treatment modifications. Frontline therapy should default to R-CHOP, with selective use of Pola-R-CHP in higher-risk profiles. In early relapse or primary refractory disease, CAR-T therapy supersedes salvage chemoimmunotherapy plus ASCT, while bispecific antibodies and targeted agents are appropriate post-CAR-T or for ineligible patients.

Moving forward, the field must focus on prospective, multi-institutional validation of emerging prognostic models, standardized PET and ctDNA thresholds, and the integration of computational approaches with clinical and molecular data. Embedding these biology-driven, risk-adapted strategies into routine practice will enable more precise patient selection and ultimately improve outcomes in DLBCL.

## Data Availability

No datasets were generated or analysed during the current study.
